# Visual Impairment Risk After Alcohol Abstinence in Patients With Newly Diagnosed Open-Angle Glaucoma

**DOI:** 10.1001/jamanetworkopen.2023.38526

**Published:** 2023-10-19

**Authors:** Yoon Jeong, Su Hwan Kim, Goneui Kang, Hyung-Jin Yoon, Young Kook Kim, Ahnul Ha

**Affiliations:** 1 Department of Ophthalmology, Seoul National University Hospital, Seoul, Korea; 2 Biomedical Research Institute, Seoul National University Hospital, Seoul, Korea; 3 EyeLight Data Science Lab, Seoul National University College of Medicine, Seoul, Korea; 4 Medical Bigdata Research Center, Seoul National University College of Medicine, Seoul, Korea; 5 Department of Ophthalmology, Seoul National University College of Medicine, Seoul, Korea; 6 Department of Ophthalmology, Jeju National University Hospital, Jeju-si, Korea; 7 Department of Ophthalmology, Jeju National University College of Medicine, Jeju-si, Korea

## Abstract

**Importance:**

Recent studies indicate that alcohol consumption is linked to increased intraocular pressure and higher prevalence of open-angle glaucoma (OAG). However, there is insufficient evidence to establish any correlation between alcohol abstinence and improved outcomes in patients with OAG.

**Objective:**

To evaluate the association between alcohol consumption status (and its changes) and risk of incident severe visual impairment (VI) or blindness in patients with newly diagnosed OAG.

**Design, Setting, and Participants:**

This retrospective, nationwide, population-based cohort study used the Korean National Health Insurance Service’s claims and health examination database to enroll patients who were newly diagnosed with OAG between January 1, 2010, and December 31, 2011, and had been alcohol drinkers before their OAG diagnosis. The cohort was followed up until December 2020. The data were analyzed from February to December 2022.

**Exposures:**

The patients were categorized into 2 groups based on their post–OAG diagnosis alcohol consumption status: sustainers and abstainers. The risks of severe VI or blindness were compared using weighted Cox proportional hazards regression models along with inverse probability of treatment weighting.

**Main Outcomes and Measures:**

Incident severe VI or blindness.

**Results:**

Among 13 643 patients with newly diagnosed OAG (mean [SD] age, 53.7 [11.9] years; 12 066 men [88.4%]) who were drinkers, 2866 (21.0%) quit drinking after the diagnosis. During 91 366 person-years of follow-up, patients abstaining from alcohol after their OAG diagnosis had a lower risk of severe VI or blindness than did those who had sustained drinking (adjusted hazard ratio [AHR] after inverse probability of treatment weighting, 0.63; 95% CI, 0.45-0.87). Among the sustained drinkers, both mild consumption (<105 g/wk; AHR, 1.52; 95% CI, 1.01-2.28) and moderate to heavy consumption (≥105 g/wk; AHR, 1.78; 95% CI, 1.11-2.86) after OAG diagnosis were associated with higher risk of severe VI or blindness relative to abstainers. Frequent drinking (≥4 d/wk) also was associated with a higher risk of severe VI or blindness (AHR, 2.56; 95% CI, 1.52-4.33) compared with abstinence.

**Conclusions and Relevance:**

In this cohort study of patients with OAG who were drinkers, abstaining from alcohol after an OAG diagnosis was associated with lower risk of severe VI or blindness. These findings suggest that lifestyle interventions, such as alcohol abstinence, could be essential for patients with newly diagnosed OAG.

## Key Points

**Question:**

Is reduced alcohol consumption or abstinence associated with the clinical outcomes of patients with open-angle glaucoma?

**Findings:**

In this population-based cohort study of 13 643 patients with newly diagnosed open-angle glaucoma, abstinence from alcohol after glaucoma diagnosis was associated with a statistically significant lower risk of severe visual impairment or blindness compared with sustained drinkers.

**Meaning:**

The findings of this study suggest that lifestyle interventions, such as attention to and curtailment of alcohol consumption, may be warranted in patients with newly diagnosed open-angle glaucoma.

## Teaser

This cohort study of patients with newly diagnosed open-angle glaucoma examines whether alcohol consumption status is associated with risk of incident severe visual impairment or blindness.

## Introduction

Glaucoma, a group of diseases characterized by progressive optic neuropathy,^1^ is the leading cause of irreversible blindness worldwide, with current estimates of 76 million cases projected to increase to 112 million by 2040.^2^ Although many genetic and environmental factors are known to be associated with glaucoma development and progression, currently the only modifiable causative risk factor is intraocular pressure (IOP).^3^ As medicine evolves toward more holistic approaches, however, it seems important to raise awareness of how modification of lifestyle can positively affect glaucoma prognosis.

Alcohol consumption is a factor in a number of chronic diseases of various organ systems and has been implicated as a leading cause of death and disability.^4^ Long-term use of alcohol has been confirmed as being associated with cardiovascular and endocrine disorders and neurodegenerative disorders.^5,6^ The underlying mechanisms proposed for these associations are oxidative stress (leading to free-radical damage to nerves), activation of sympathoadrenal and hypothalamic- pituitary-adrenal axes, nutritional deficiencies, and direct toxic and proinflammatory effects.^7^

Recent studies show that alcohol consumption is associated with both elevated IOP and higher prevalence of open-angle glaucoma (OAG).^8,9^ A linear dose-response relationship of alcohol intake with glaucoma-related outcomes (ie, higher IOP, thinner retinal nerve fiber layer, and higher OAG prevalence) also has been reported.^8^ These findings suggest that nondrinkers or mild drinkers are less likely to develop glaucoma and to have a more positive prognosis than are heavy drinkers.

However, there has been no confirmatory evidence of an association between alcohol abstinence after OAG diagnosis and better outcomes relative to those for sustained drinkers. Given that alcohol consumption–based randomized clinical trials are not ethically appropriate, well-designed epidemiologic studies remain the most effective way to investigate the importance of alcohol abstinence. In the present study, we evaluated, in a nationwide, population-based cohort, the association between alcohol consumption status (and its changes) after initial OAG diagnosis and risk of incident severe visual impairment (VI) or blindness.

## Methods

We used the claim database and health screening data from the Korean National Health Insurance (NHIS) for this cohort study. South Korea has a mandatory health insurance system for all Korean residents and health care practitioners. In 2019, the NHIS covered 97.2% of the population, including employee-insured and self-employed–insured individuals, and the medical aid beneficiaries system covered the remaining 2.8% of the population. Therefore, the NHIS database contains records for approximately the entire 50 million Korean residents.^10^ The database includes enrollees’ demographics and medical diagnostic records coded according to the *International Statistical Classification of Diseases and Related Health Problems, Tenth Revision (ICD-10)*, along with information on examinations, prescriptions, and procedures. The NHIS offers a free biennial national health screening to all enrollees 20 years or older; the examination includes a self-report questionnaire for evaluation of health-related behaviors, including smoking, alcohol consumption, and exercise, among others. This project was approved by the institutional review board of Seoul National University Hospital, and the need for informed consent was waived because the data had been anonymized and were accessed at a closed-network onsite location. This study was conducted in accordance with the Declaration of Helsinki^11^ and designed according to the Strengthening the Reporting of Observational Studies in Epidemiology (STROBE) reporting guideline.

### Design and Participants

**Figure 1.**
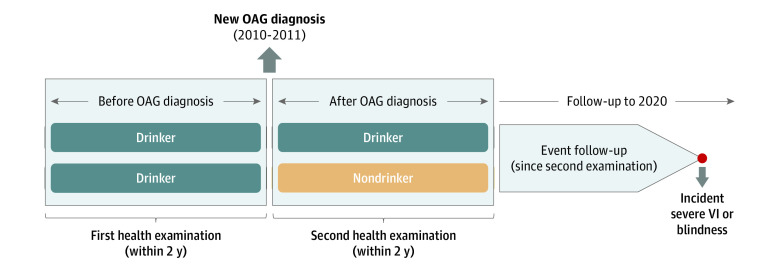
Study Design OAG indicates open-angle glaucoma; VI, visual impairment.

Figure 1 and the eFigure in Supplement 1 present, respectively, the study design and a flowchart of participant enrollment. We included patients who had been newly diagnosed with OAG between January 1, 2010, and December 31, 2011 (n = 262 343). The OAG diagnosis was confirmed only when a patient had been assigned an OAG code (H40.1) and prescribed 1 or more antiglaucoma medications for more than 3 months or had undergone glaucoma surgery. We set a washout period, excluding any patients who had been diagnosed with OAG within the preceding 2 years (2008-2009).

We then selected patients who had undergone 2 separate health examinations: a first (baseline) examination within the 2-year period before the OAG diagnosis date and a second examination within the 2-year period after diagnosis. The OAG diagnosis date for each patient was defined as the date on which the H40.1 code had first been entered or the date antiglaucoma medication had first been prescribed, whichever came first.

We excluded patients for whom values were missing from the health examination data. Patients who had been diagnosed with 2 other major sight-threatening eye diseases that can potentially cause severe VI, namely, exudative age-related macular degeneration and diabetic retinopathy, were also excluded. To ensure the accuracy of our recorded primary outcome, we excluded patients who already were severely visually impaired or blind before the second health examination date.

### Exposure

**Figure 2.**
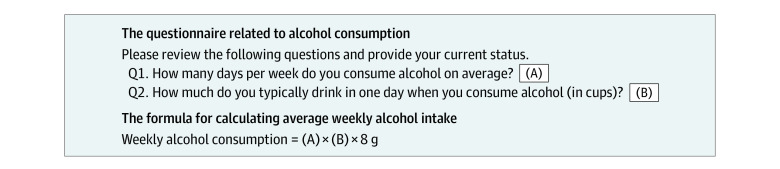
Questionnaire Regarding Alcohol Consumption for the Korean Nationwide Health Examination (Translated Into English)

Alcohol consumption status and changes to it were evaluated based on a self-reported questionnaire. Patients who were drinkers at the time of the first health examination were categorized into 1 of 2 groups based on their status at the second health examination: sustained drinkers or abstainers from alcohol after OAG diagnosis. We calculated the pure alcohol intake per week with the following information: drinking frequency per week, amount consumed on each occasion, and typical alcohol content in standard drink (Figure 2; eMethods in Supplement 1).^12^ We divided the participants by post–OAG diagnosis drinking level according to the Dietary Guidelines for Americans^13^: mild (<105 g/wk) or moderate to heavy (≥105 g/wk). Additionally, drinking frequency per week was categorized as less frequent (≤3 d/wk) or regular to frequent (≥4 d/wk).

### Outcomes, Follow-Up, and Covariates

The primary outcome was incidence of severe VI or blindness during follow-up. We used both the NHIS Screening Program databases and the National Handicapped Registry for coexisting VI. A detailed description on VI assessment is given in the eMethods in Supplement 1. The index date of follow-up was defined as the date of the second health examination, which was performed within 2 years of the OAG diagnosis. Patients were monitored from the index date until the onset of severe VI or blindness, death, or the study end date (December 31, 2020), whichever came first. Comorbidities included hypertension, diabetes, dyslipidemia, chronic kidney disease, chronic obstructive pulmonary disease, and cancer. Detailed definitions are presented in eTable 1 in Supplement 1.

### Statistical Analysis

Analyses were conducted between February and December 2022. Continuous variables were recorded as means (SDs) and categorical variables as numbers (percentages). One-way analysis of variance and the χ^2^ test were used to evaluate the significance of the differences between the groups as categorized by alcohol consumption status. The severe VI or blindness incidence rate was calculated by dividing the number of incident cases by the total follow-up period and was recorded as per 100 000 person-years. The adjusted hazard ratio (AHR) and 95% CI for severe VI or blindness were analyzed according to the Cox proportional hazards regression model.

We compared sustained drinkers and abstainers using inverse probability of treatment weighting (IPTW) analysis,^14,15^ which has an advantage over propensity score matching in that it retains more individuals (thus increasing its power) and estimates hazards with less bias.^16^ The propensity for being in a group was calculated based on a logistic regression model with baseline covariates that included age, sex, comorbidities, body mass index, smoking status, regular exercise, and low income.^17^ To evaluate the intergroup balance of baseline characteristics, the absolute standardized difference was calculated for each of the covariates. An absolute standardized difference of 0.1 or less indicated a negligible covariate difference between the 2 groups.^18^ The risk of severe VI or blindness in both groups was obtained by means of weighted Cox proportional hazards regression models with IPTW.

Sensitivity analyses were conducted to investigate whether the interaction of specific treatments, such as glaucoma medications or surgical procedures, with alcohol had any influence on the results. A 2-sided *P* < .05 was considered to signify statistical significance. Data analyses were performed using SAS statistical software, version 9.4 (SAS Institute Inc), and plots were generated using R software, 4.0.0 (R Foundation for Statistical Computing).

## Results

A total of 13 643 alcohol-consuming patients with newly diagnosed OAG (mean [SD] age, 53.7 [11.9] years; 12 066 men [88.4%] and 1577 [11.6%] women) were included. The mean (SD) interval between the baseline health examination and OAG diagnosis was 1.5 (1.2) years and between OAG diagnosis and the second health examination was 1.9 (1.4) years.

In the total study population, 2866 (21.0%) abstained from alcohol after OAG diagnosis, and 10 777 (79.0%) sustained drinking. Among the sustained drinkers, 7458 (69.2%) were mild drinkers and 3319 (30.8%) were moderate to heavy drinkers. Compared with the abstainer group, sustainers tended to be younger and male and had a lower prevalence of most comorbidities. The sustained drinker group had a larger proportion of smokers as well as a lower proportion of low-income members (Table 1).

**Table 1.** Baseline Characteristics Before and After IPTW^a^

Characteristic	Before IPTW			After IPTW		
	Abstainers (n = 2866)	Sustained drinkers (n = 10 777)	Maximum ASD	Abstainers (n = 21 599)	Sustained drinkers (n = 17 450)	Maximum ASD
Age, y	57.3 (12.7)	52.9 (11.7)		57.8 (39.4)	54.8 (15.7)	
≤40	297 (10.4)	1576 (14.6)	0.365	2369 (11.0)	2215 (12.7)	0.098
41-64	1650 (57.6)	7249 (67.3)		11 434 (52.9)	11 038 (63.3)	
≥65	919 (32.1)	1952 (18.1)		7795 (36.1)	4197 (24.1)	
Sex						
Male	2164 (75.5)	9902 (91.9)	0.455	18 337 (84.9)	14 735 (84.4)	0.013
Female	702 (24.5)	875 (8.1)		3262 (15.1)	2715 (15.6)	
Comorbidities						
Hypertension	1669 (58.3)	6187 (57.4)	0.017	13 130 (60.1)	10 059 (57.6)	0.064
Diabetes	434 (15.1)	1526 (14.2)	0.028	3610 (16.7)	2529 (14.5)	0.061
Dyslipidemia	296 (10.3)	1076 (10.0)	0.011	1977 (9.2)	1759 (10.1)	0.031
COPD	410 (14.3)	1059 (9.8)	0.138	3196 (14.8)	2058 (11.8)	0.088
CKD	275 (9.6)	509 (4.7)	0.190	1996 (9.2)	1209 (6.9)	0.085
Cancer	97 (3.4)	163 (1.5)	0.121	621 (2.9)	402 (2.3)	0.036
BMI	23.9 (3.0)	24.3 (2.9)	0.136	23.9 (8.9)	24.1 (3.7)	0.029
Smoker	1307 (45.6)	7653 (71.0)	0.377	9052 (42.0)	10 487 (60.1)	0.262
Regular exercise	402 (14.0)	1644 (15.3)	0.024	3345 (15.5)	2546 (14.6)	0.018
Low income	684 (23.9)	1810 (16.8)	0.125	5468 (25.3)	3460 (19.8)	0.093

Abbreviations: ASD, absolute standardized difference; BMI, body mass index (calculated as weight in kilograms divided by height in meters squared); CKD, chronic kidney disease; COPD, chronic obstructive pulmonary disease; IPTW, inverse probability of treatment weighting.

^a^ Continuous variables are presented as mean (SD) and categorical variables as number (percentage).

### Risk of VI According to Alcohol Consumption Status After OAG Diagnosis

During the 91 366 person-years of follow-up, 58 patients with OAG were diagnosed with incident severe VI or blindness (64 per 100 000 person-years). Among the 58 patients, 45 were alcohol sustainers, whereas the remaining 13 were abstainers. After IPTW, most of the baseline characteristics between the 2 groups were well balanced (Table 1). In a multivariable-adjusted Cox proportional hazards regression model analysis, patients abstaining from alcohol after their OAG diagnosis were associated with a lower risk of severe VI or blindness relative to the sustained drinkers (AHR, 0.63; 95% CI, 0.45-0.87) (Table 2).

**Table 2.** Adjusted Hazard Ratios (AHRs) for Severe Visual Impairment or Blindness After Inverse Probability of Treatment Weighting According to the Alcohol Consumption Status

Alcohol consumption status	No. of patients	No. of events	Crude IR	AHR (95% CI)^a^
Current alcohol intake				
Abstainers	21 599	71	46.8	0.63 (0.45-0.87)
Sustained drinkers	17 450	81	65.3	1 [Reference]
Amount of alcohol consumed per week				
Abstainers	21 599	71	46.8	1 [Reference]
Mild (0-104 g)	12 077	51	59.5	1.52 (1.01-2.28)
Moderate to heavy (≥105 g)	5373	30	78.4	1.78 (1.11-2.86)
No. of drinking session per week				
Abstainers	21 599	71	46.8	1 [Reference]
Less frequent (1-3 d)	15 466	64	57.9	1.42 (0.97-2.08)
Regular to frequent (4-7 d)	1985	17	123.2	2.56 (1.52-4.33)
Reduction rate of alcohol intake^b^				
Great (>66%)	25 123	92	52.0	1 [Reference]
Moderate (≤66% to >33%)	3114	12	53.3	1.09 (0.59-1.98)
Small (≤33% to >0%)	2741	10	49.2	1.23 (0.63-2.40)
No change or increase (≤0%)	8071	38	67.3	1.34 (0.90-2.01)

Abbreviation: IR, incidence ratio (per 100 000 person-years).

^a^ Hazard ratios were adjusted for age, sex, baseline body mass index, smoking, regular exercise, low income, and comorbidities including hypertension, diabetes mellitus, dyslipidemia, chronic kidney disease, chronic obstructive pulmonary disease, and cancer.

^b^ Rate of changes in alcohol consumption amount between first examination (before open-angle glaucoma diagnosis) and second examination (after open-angle glaucoma diagnosis).

The sensitivity analyses indicated that patients with OAG who had received glaucoma surgery were at higher risk of VI if they did not reduce alcohol consumption; however, the interaction effect was not evident. In addition, no apparent interaction effect with alcohol was observed for topical medication (eTable 2 in Supplement 1).

### Risk of VI According to Alcohol Consumption Amount After OAG Diagnosis

**Figure 3.**
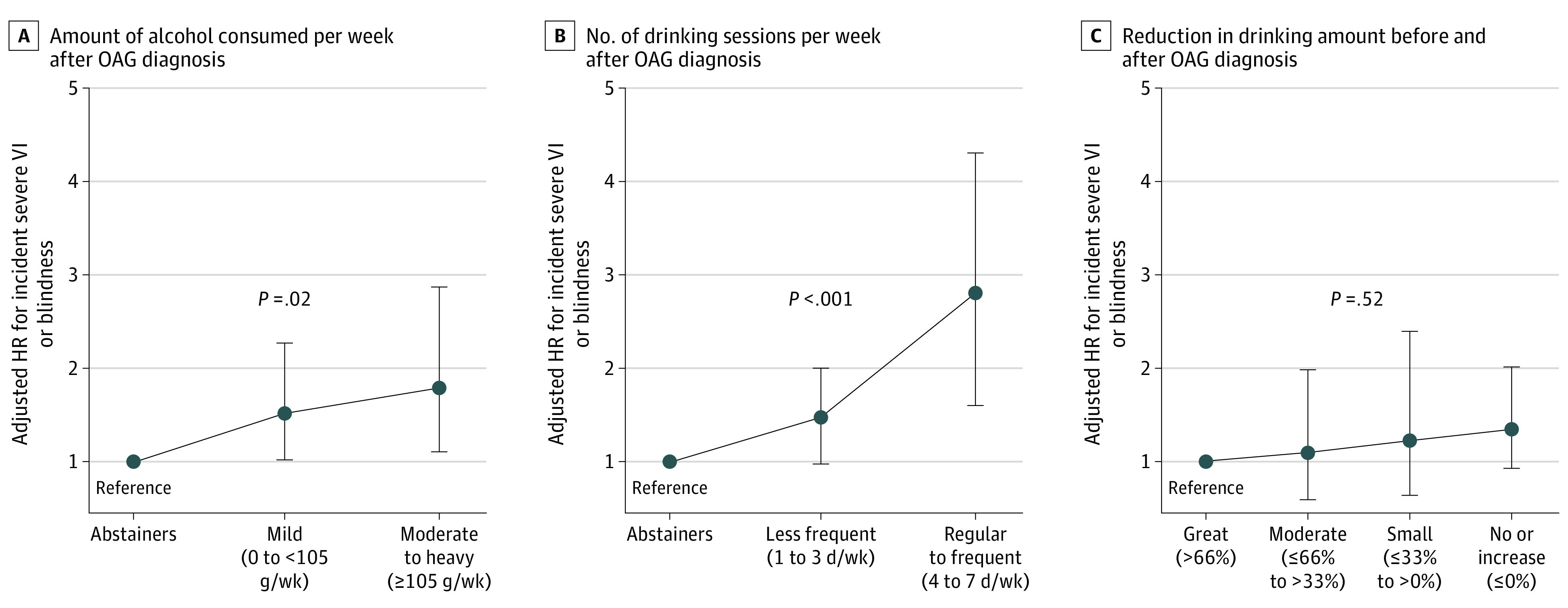
Risk of Incident Severe Visual Impairment (VI) or Blindness According to Alcohol Consumption Status and Its Changes Hazard ratios (HRs) were adjusted for age, sex, baseline body mass index, smoking, regular exercise, low income, and comorbidities (ie, hypertension, diabetes, dyslipidemia, chronic kidney disease, chronic obstructive pulmonary disease, and cancer). OAG indicates open-angle glaucoma. Whiskers represent 95% CIs.

Mild consumption (<105 g/wk) showed a significantly higher risk of severe VI or blindness than did abstinence (AHR, 1.52; 95% CI, 1.01-2.28), and moderate to heavy consumption (≥105 g/wk) was associated with an even higher risk (AHR, 1.78; 95% CI, 1.11-2.86) (Table 2). Higher alcohol consumption was therefore associated with higher risk of severe VI or blindness (*P* = .02) (Figure 3A).

### Risk of VI According to Number of Drinking Sessions After OAG Diagnosis

Higher frequency of weekly drinking after OAG diagnosis was a significant risk factor for incident severe VI or blindness (*P* < .001) (Figure 3B). Regular to frequent consumption (≥4 days) presented a significantly higher risk of severe VI or blindness compared with abstinence (AHR, 2.56; 95% CI, 1.52-4.33) (Table 2). Less frequent drinkers (≤3 days) exhibited an elevated risk of severe VI or blindness, although this association did not achieve statistical significance (AHR, 1.42; 95% CI, 0.97-2.08).

### Risk of VI According to Changes of Drinking Amount Before and After OAG Diagnosis

The total study population was stratified by extent of alcohol intake reduction after OAG diagnosis (great reduction [>66%], moderate reduction [≤66% to >33%], small reduction [≤33% to >0%], or no reduction or increased consumption [≤0%]). No significant associations between the degree of alcohol consumption reduction and the risk of severe VI or blindness was identified (Figure 3C).

## Discussion

This study investigated, in a nationwide, population-based cohort of patients with newly diagnosed OAG, the association between alcohol consumption status (and its changes) and risk of incident severe VI or blindness. The following are our principal findings: (1) a significant proportion (79%) of patients with newly diagnosed OAG remained current drinkers; (2) abstinence from alcohol after OAG diagnosis was associated with lower risk of severe VI or blindness relative to sustained drinkers; (3) persistent drinkers with any alcohol intake amount (even mild intake) incurred a higher risk of severe VI or blindness relative to abstainers; and (4) heavier as well as more frequent drinkers incurred higher risk.

Recently, a meta-analysis summarizing 34 studies identified significant associations between alcohol consumption and both higher IOP and greater OAG risk.^9^ In addition, a large population-based study based on UK Biobank data showed strong as well as consistent adverse dose-response associations between consumption of alcohol and all glaucoma-related outcomes (eg, IOP, retinal nerve fiber layer or ganglion cell inner plexiform layer thickness, and OAG prevalence).^8^ However, for active encouragement of and education on alcohol abstinence, more robust evidence is needed on whether reducing alcohol consumption after OAG diagnosis could impact glaucoma’s clinical course.

Analyzing a nationwide cohort, our study found that quitting alcohol after newly diagnosed OAG was associated with a 37% lower risk of severe VI or blindness compared with continued consumption. A significant proportion of Korean patients with OAG (up to 77%) have normal IOP levels.^19^ Given the inherently limited therapeutic capacity of IOP reduction among patients already in the normotensive IOP range, identifying modifiable factors beyond IOP becomes crucial.^20^ Apart from alcohol cessation, other lifestyle interventions, such as aerobic exercise, stress reduction through mindfulness, and a nutrient-rich diet, have been proposed to lower IOP and/or provide neuroprotection.^21^ However, longer studies are needed to confirm and assess the sustainability of these effects. Notably, in Korea, alcohol consumption is substantial, averaging 10.2 L per capita in 2016.^22^ This figure surpasses the World Health Organization Western Pacific Region mean of 7.3 L.^22^ However, alcohol guidelines remain unclear in Korea. Our results emphasize the need to integrate alcohol awareness and cessation into a comprehensive strategy for patients with newly diagnosed OAG.

There are a significant number of biological mechanisms plausibly underlying the associations observed in this study. It is well established that alcohol has neurotoxic properties that, with habitual and/or heavy consumption, can be associated with decreased brain volume,^23^ peripheral neuropathy,^7^ and neurodegenerative disorders, such as Alzheimer and Parkinson diseases.^24^ This in fact may be a major etiologic factor, particularly given that the retina is an extension of the central nervous system. Indeed, previous studies have consistently found thinner retinal thickness to be associated with long-term alcohol intake.^25,26^

Oxidative stress–mediated damage to the trabecular meshwork might account for the IOP elevation that is associated with cumulative alcohol consumption and may further contribute to worse clinical courses and outcomes through the usual IOP-dependent mechanisms.^27^ Moreover, indirect effects, such as from detrimental cardiovascular diseases (eg, hypertension and atherosclerosis) incurred through heavy drinking, may have implications with regard to glaucomatous neurodegeneration occurring via IOP-independent mechanisms.^28,29,30^ In addition, it is plausible that the beneficial effects of alcohol abstinence actually represent a combination of factors, not just a single mechanism.

Despite the predominantly harmful health associations, alcohol exhibits a J-shaped association with certain cardiovascular outcomes, with a protective effect having been observed at low intake levels.^4^ Alcohol’s short-term effects on the human eye include a transient and seemingly dose-dependent reduction in IOP as well as increased blood flow to the optic nerve head,^31,32,33,34^ conferring, at least theoretically, a protective effect against glaucomatous damage. However, in the current study, even mild intake (<105 g/wk) had an association with worse visual outcome. This might be attributable to alcohol’s detrimental effects on glaucoma overriding any potential short-term properties that otherwise would be beneficial.

The positive effects of light alcohol consumption on coronary heart disease have also been attributed to the sick quitter effect.^35^ This phenomenon is defined as individuals reducing a hazardous activity because of health concerns. As people age, become ill, or increase their medication use, they tend to decrease their alcohol intake.^36^ Our study may not have captured all medical comorbidities or health issues that lead to alcohol cessation. Although we did not find any difference in health checkup rates based on the presence of an OAG diagnosis, the potential for introducing bias into this study, arising from underlying health conditions or a low rate of health care engagement among those who did not participate in the health checkup, should be taken into account.

### Limitations

This study has some limitations. First, the OAG diagnoses were based on claims data, and it is difficult to validate the accuracy of diagnoses using medical records. Although we rigorously defined the inclusion criteria for OAG diagnosis to improve specificity—encompassing diagnostic codes, medication prescriptions (spanning a 3-month period), and a record of glaucoma surgery—we acknowledge that diagnoses relying on claims data could be susceptible to challenges related to misclassification. Moreover, the requirement for a glaucoma diagnosis for reimbursement reasons might introduce potential biases. Second, alcohol intake could influence comorbidities, such as diabetes and obesity,^37,38^ which could also be modifiable risk factors in independent association with VI. Even after IPTW, smoking status remained incompletely balanced between current drinkers and nondrinkers. The genetic association and coexistence between alcohol and smoking has been well recognized. To overcome this limitation, we adjusted for comorbidities and smoking status; nonetheless, residual confounding remains a possibility. Third, the lack of detailed information on potential confounders, such as clinical examination data, disease severity, and health behaviors, is an inherent limitation of a claims database study. In particular, factors such as meditation and diet (including supplements and caffeine), which we did not account for, might be associated with the risk of severe VI or blindness. In addition, integrating changes in these factors into our analysis proved difficult. This acknowledgment is crucial because of the possibility that patients abstaining from alcohol might undergo other lifestyle changes concurrently. Although we accounted for major sight-threatening eye diseases, it is crucial to acknowledge the possibility that other conditions may have influenced vision and thus should be duly considered. Fourth, exposure ascertainment via self-reported alcohol consumption on a questionnaire is subject to both recall and social desirability bias and, as such, can incur misclassification. This measure might not accurately reflect alcohol consumption during a lifetime or even specific drinking patterns. However, we believe that the presence of systemic misclassification bias would not necessarily negate any observed associations, although it may have implications for quantifying degrees of risk. Fifth, although our study reinforces the recommendation of abstaining from alcohol for individuals with OAG, it did not establish alcohol as a cause of worsening glaucoma or quitting alcohol as a means of delaying its progression. Additional research with various study designs is crucial to investigations of the relationship between alcohol consumption and development or advancement of OAG.

## Conclusions

In this cohort study of patients with OAG who were drinkers, alcohol abstinence was associated with diminished risk of severe VI or blindness. Lifestyle interventions, such as alcohol abstinence, could be essential in a comprehensive approach for patients with newly diagnosed OAG.

## Notes

Supplement 1. **eMethods.** Supplemental Methods **eTable 1.** Definitions of Covariates and Clinical Outcome **eTable 2.** Hazard Ratios for Severe Visual Impairment or Blindness in Abstainers and Sustainers According to Glaucoma Surgery or Medication **eFigure.** Flowchart of Enrollment of Study Population

Supplement 2.
Data Sharing Statement
