# Supplementary material for: Visual Impairment Risk After Alcohol Abstinence in Patients With Newly Diagnosed Open-Angle Glaucoma
Source: JAMA Netw Open. 2023 Oct 19;6(10):e2338526. doi: 10.1001/jamanetworkopen.2023.38526 (PMC10587786; doi:10.1001/jamanetworkopen.2023.38526)
Supplement: Supplement 1. — eMethods. Supplemental Methods eTable 1. Definitions of Covariates and Clinical Outcome eTable 2. Hazard Ratios for Severe Visual Impairment or Blindness in Abstainers and Sustainers According to Glaucoma Surgery or Medication eFigure. Flowchart of Enrollment of Study Population [file jamanetwopen-e2338526-s001.pdf]

Supplementary Online Content

Jeong Y, Kim SH, Kang G, Yoon H-J, Kim YK, Ha A. Visual impairment risk after alcohol abstinence in patients with newly diagnosed open-angle glaucoma. *JAMA Netw Open*. 2023;6(10):e2338526. doi:10.1001/jamanetworkopen.2023.38526

eMethods. Supplemental Methods

eTable 1. Definitions of Covariates and Clinical Outcome

eTable 2. Hazard Ratios for Severe Visual Impairment or Blindness in Abstainers and Sustainers According to Glaucoma Surgery or Medication

eFigure. Flowchart of Enrollment of Study Population

This supplementary material has been provided by the authors to give readers additional information about their work.

© 2023 Jeong Y et al. *JAMA Network Open.*

eMethods. Supplemental Methods

Calculation of Amount of Alcohol Consumption

The questionnaire regarding alcohol consumption for the Korean nationwide health examination is as follows (translated into English).

Please read the following questions and fill out your current situation.<br>On average, how many days a week do you drink?<br>□0 □1 □2 □3 □4 □5 □6 □7<br><br>How much do you usually drink a day when you drink (cup)? ________

In South Korea, a cup of beer contains 220 ml of beer, and a cup of Soju (Korean traditional alcohol beverage) contains 50 ml of Soju. We assumed that beer contains 4.5% of alcohol, a Soju contains 21.0% of alcohol, and the specific gravity of alcohol is 0.79. According to these assumption, a cup of beer contains 7.8 g of alcohol (220 * 0.045 * 0.79 = 7.821) and a cup of Soju contains 8.3 g of alcohol (50 * 0.21 * 0.79 = 8.295). Therefore, we assumed that a standard drink (one cup for each alcohol type) contains 8 g of alcohol.[1] Although we calculated the amount of alcohol consumption based on beer and Soju because the majority of alcohol consumption in Korea was based on beer and Soju, in contrast to western countries according to the data from the Korean Customs Service and Korean Alcohol Liquor Industry Association (http://www.kalia.or.kr), other alcohol types such as whiskey and wine have their own glass which contains a similar amount of alcohol compared with our calculation based on beer and Soju.

Assessment of Visual Impairment

We utilized both the NHIS Screening Program databases and the National Handicapped Registry for coexisting VI. If the better eye's corrected visual acuity was < 6/60 in the health checkup record, severe VI or blindness was recorded. In Korea, in order to be registered as legal severe VI in the National Handicapped Registry, a patient must submit an ophthalmologist-issued medical certificate concerning the best-corrected visual acuity (BCVA), the visual field, and the probable cause of VI. Legal severe VI is defined as any of the following two conditions showing stabilization after six months or more of treatment and not reversed by either medication or surgery (excepting keratoplasty): 1) BCVA ≤ 6/100 in better eye or 2) visual field ≤ 5 degrees from visual axis for both eyes.

Reference

1. Kim YH, Han KD, Choi JI, *et al*. Frequent drinking is a more important risk factor for new-onset atrial fibrillation than binge drinking: a nationwide population-based study. *Europace* 2020;22:216-224.

© 2023 Jeong Y et al. *JAMA Network Open.*

eTable 1. Definitions of Covariates and Clinical Outcome

Diagnosis	ICD-10 code and definition	Diagnostic definition
**Inclusion criteria**		
**Open-angle glaucoma**	H40.1; and minimum 1 prescription of anti-glaucoma medication for more than 3 months or had received glaucoma surgery.	Outpatient department≥2
**Exclusion criteria**		
**Exudative AMD**	V201 (identified using the registration program database for rare intractable diseases)	Outpatient department≥2
**DR**	H36.0, H36.8; and minimum 1 prescription of anti-diabetic drugs (sulfonylureas, metformin, meglitinides, thiazolidinediones, dipeptidyl peptidase-4 inhibitors, α-glucosidase inhibitors, and insulin).	Admission≥1 or outpatient department≥2
**Comorbidities**		
**Hypertension**	I10-I13, I15; and minimum 1 prescription of anti-hypertensive drug (thiazide, loop diuretics, aldosterone antagonist, alpha-/beta-blocker, calcium-channel blocker, angiotensin-converting enzyme inhibitor, angiotensin II receptor blocker).	Admission≥1 or outpatient department≥2
	or systolic/diastolic blood pressure ≥ 130/80 mmHg	Based on the results of 2nd health exam
**Diabetes mellitus**	E11-E14; and minimum 1 prescription of anti-diabetic drugs (sulfonylureas, metformin, meglitinides, thiazolidinediones, dipeptidyl peptidase-4 inhibitors, α-glucosidase inhibitors, and insulin).	Admission≥1 or outpatient department≥2
	or fasting glucose level ≥ 126 mg/dL	Based on the results of 2nd health exam
**Dyslipidemia**	E78	Admission or outpatient department≥1
	or Total cholesterol ≥ 240 mg/dL	Based on the results of 2nd health exam
**CKD**	eGFR<60ml/min/1.73m^2	Based on the results of 2nd health exam
**COPD**	J41-44	Admission or outpatient department≥1
**Cancer**	C00-97 and rare intractable diseases code (V193)	Admission or outpatient department≥1
**Health exam questionnaire**		
**Alcohol consumption (weekly alcohol intake amount)**	Non (0 g)<br>Mild (0< g <105)<br>Moderate-to-heavy (105 ≤ g)	Based on the results of 2nd health exam
**Smoking**	Never smoker	Based on the results of 2nd health exam

© 2023 Jeong Y et al. *JAMA Network Open.*

	Ex-smoker	
	Current smoker	
**Regular exercise**	Performing a moderate physical activity more than 30 minutes at least 5 times per week or strenuous physical activity more than 20 minutes at least 3 times per week.	Based on the results of 2nd health exam
**Low income**	Income lowest 20% and medical aid	

ICD, International Classification of Diseases; AMD, age-related macular degeneration; DR, diabetic retinopathy; CKD, chronic kidney disease; COPD, chronic obstructive pulmonary disease; eGFR, estimated glomerular filtration rate.

© 2023 Jeong Y et al. *JAMA Network Open.*

eTable 2. Hazard Ratios for Severe Visual Impairment or Blindness in Abstainers and Sustainers According to Glaucoma Surgery or Medication

Current alcohol intake	Number	Events	Crude IR	Adjusted HR* (95% CI)	Adjusted HR** (95% CI)	p-value***
**Glaucoma surgery**						
Abstainers without surgery	20805	61	41.78	0.11 (0.07, 0.19)	0.64 (0.45, 0.91)	0.29
Sustained drinkers without surgery	16977	70	57.97	0.18 (0.10, 0.30)		
Abstainers with surgery	993	10	141.67	0.28 (0.11, 0.72)	0.28 (0.11, 0.72)	
Sustained drinkers with surgery	648	18	387.21	1 (reference)		
**Glaucoma medication**						
Abstainers without beta blocker	7847	18	31.77	0.36 (0.20, 0.60)	0.99 (0.46-2.11)	0.20
Sustained drinkers without beta blocker	6868	11	23.35	0.36 (0.19, 0.68)		
Abstainers with beta blocker	13752	54	55.38	0.56 (0.39-0.83)	0.56 (0.39-0.83)	
Sustained drinkers with beta blocker	10582	69	92.67	1 (reference)		

*Adjusted HR: adjusted for age, sex, baseline body mass index, smoking, regular exercise, low income, comorbidities including hypertension, diabetes mellitus, dyslipidemia, chronic kidney disease, chronic obstructive pulmonary disease and cancer.
**Adjusted HR for alcohol abstinence after adjusting for surgery or medication.
***p-value for interaction term between surgery/medication and alcohol abstinence
IR, incidence ratio (per 100,000 person-years); HR, hazard ratio; CI, confidence interval.

© 2023 Jeong Y et al. *JAMA Network Open.*

eFigure 1. Flowchart of Enrolment of Study Population. OAG, open-angle glaucoma; VI, visual impairment

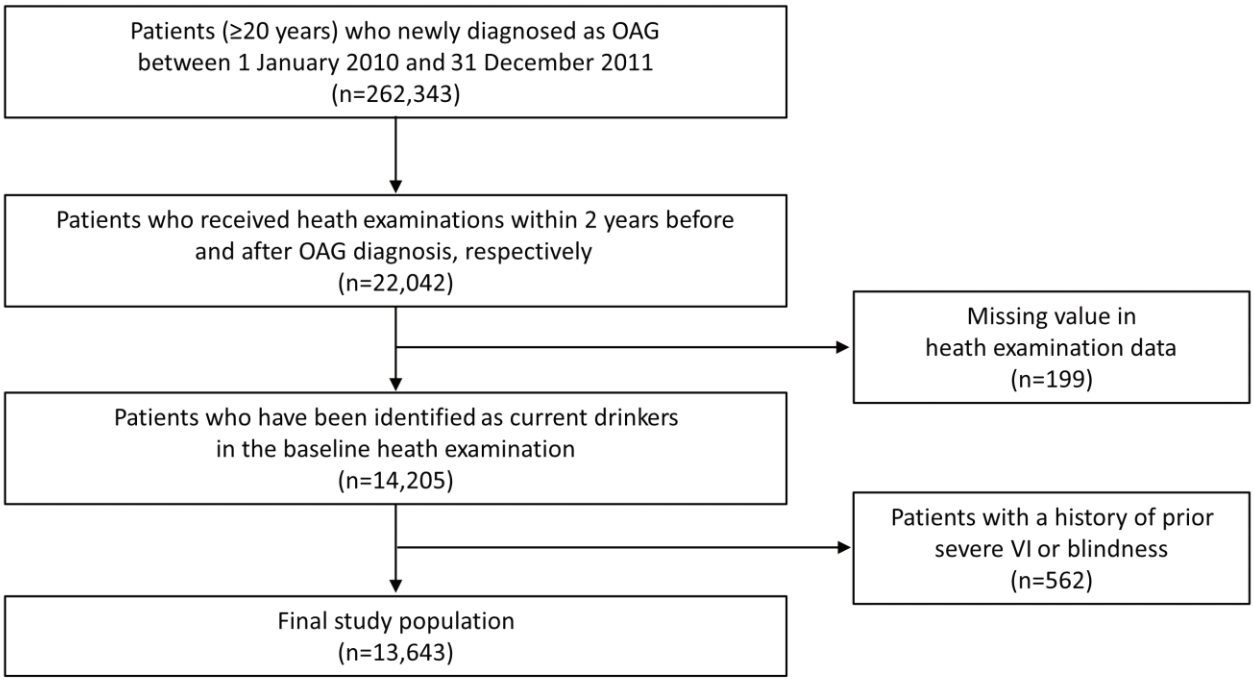

© 2023 Jeong Y et al. *JAMA Network Open.*
