# Supplementary material for: Visual Impairment Risk After Alcohol Abstinence in Patients With Newly Diagnosed Open-Angle Glaucoma
Source: JAMA Netw Open. 2023 Oct 19;6(10):e2338526. doi: 10.1001/jamanetworkopen.2023.38526 (PMC10587786; doi:10.1001/jamanetworkopen.2023.38526)
Supplement: Supplement 2. — Data Sharing Statement [file jamanetwopen-e2338526-s002.pdf]

Data Sharing Statement

Jeong. Visual Impairment Risk After Alcohol Abstinence in Patients With Newly Diagnosed Open-Angle Glaucoma. *JAMA Netw Open*. Published October 19, 2023.
doi:10.1001/jamanetworkopen.2023.38526

Data
Data available: Yes
Data types: Deidentified participant data
How to access data: All data analyzed during the sustained study are available at the National Health Insurance Data Sharing Service (accessed at https://nhiss.nhis.or.kr/bd/ab/bdaba000eng.do).
When available: With publication

Supporting Documents
Document types: None

Additional Information
Who can access the data: researchers whose proposed use of the data has been approved.
Types of analyses: for an academic purpose.
Mechanisms of data availability: after approval of a proposal.
